# Unity in diversity: mapping healthcare interpreting studies (2007-2017)

**DOI:** 10.1080/10872981.2019.1579559

**Published:** 2019-03-08

**Authors:** Yubo Liu, Wei Zhang

**Affiliations:** a Graduate School of Translation and Interpretation, Beijing Foreign Studies University, Beijing, China; b School of English and International Studies, Beijing Foreign Studies University, Beijing, China

**Keywords:** Healthcare interpreting, mental health, healthcare interpreters, language access, bibliometric studies

## Abstract

This paper intends to examine the evolution of healthcare interpreting studies published in SSCI and A&HCI journals between 2007 and 2017. A total of 40 journal articles have been selected from 23 SSCI or A&HCI journals, covering journals on translation and interpreting, linguistics and communication as well as healthcare studies. Thematically, articles selected fall in such four major types as socio-political background, healthcare interpreting practice, and education and training. Methodologically, based on an existing framework of classification, articles selected are grouped into four categories, namely, pure empirical research, pro-empirical research, pro-non-empirical research, and pure non-empirical research. Built on such a characterization of past studies on healthcare interpreting, this paper serves as a general map of relevant studies of this sub-field in the past decade and attempts to provide recommendations on research directions in the future. Such directions include the integration and alignment between the T & I community and the healthcare research community in the study of healthcare interpreting, the development of inter-disciplinary perspectives on the socio-political background of healthcare interpreting, and the expansion of research that is specialty-based, informed and driven.

## Introduction

As a young discipline, interpreting studies have enjoyed burgeoning development in the last decade, the bulk of such development being propelled by the swift rise of community interpreting, or public service interpreting, which has risen to remarkable prominence thanks to, among other factors, the surge of immigration, a trend that has been keenly felt in Europe, North America and the Asia–Pacific. In community interpreting studies, different settings for interpreting have been examined by scholars, including legal interpreting, healthcare interpreting, educational interpreting, faith-related interpreting [1], police interpreting, asylum interpreting, etc. Among them, healthcare interpreting stands out as a major component of community interpreting. However, this sub-field of interpreting studies have not been systematically reviewed and analyzed, resulting in a lack of transparency and visibility regarding the research themes and methods on the micro level and the socio-political and socio-cultural backgrounds of such research spanning extensive geographic and demographic constituencies on a macro level, making it difficult to identify problems and weaknesses in the current state and offer suggestions for future directions. Therefore, it is the purpose of this paper to map the advances and problems of healthcare interpreting studies over the past decade and put forward directions and suggestions on future studies in this field.

## Literature review

There are several reasons for the very fact that healthcare interpreting occupies an important position in the general domain of public service interpreting or dialogue interpreting, with unique features distinguishing it apart from other sub-disciplines. First, the development of healthcare interpreting has been a result of legal progress mainly in European countries and North America, embedding this profession and its practice, from the moment of its own inception, within the socio-political and socio-cultural context of respective countries. It is exactly because of this close marriage between healthcare interpreting as a practice and the macro-level legal, political, cultural and economic environment that this profession demonstrates varying degrees of maturity in different contexts. While the US is widely acknowledged as the country most proactive in state and federal efforts in ensuring the rights and interests of patients with limited English proficiency to access interpreting services in healthcare settings [2], European countries have experienced more fluctuations in the level of government support [3,4]. Second, the venue of healthcare interpreting does make a difference, since venues constitute one of the determining factors in ‘the purpose of the communication, the context of the communication, and the consequences of miscommunication’ [5]. In a typical healthcare setting, all three parties are engaged in a collaborative attempt to achieve the optimal outcome from consultations, and any misunderstanding would, in the worst scenarios, be fatal [6]. The paramount significance attached to the consequences of interpreter-mediated healthcare encounters means that utmost caution should be taken by both service providers and interpreters. Third, healthcare interpreting encompasses a wide range of specialties, sub-types, and modalities, all of which entail their own constellations of norms and standards. Some specialties, mental health, for example, may require drastically different expertise from both service providers and interpreters, as compared with physical specialties [7]. Thus, specialty specificity constitutes a major characteristic of healthcare interpreting.

Given the above-mentioned features of healthcare interpreting that distinguish this sub-discipline apart from others, healthcare interpreting researchers inevitably face a series of unique complexities and challenges. First, the inter-disciplinary nature of healthcare interpreting studies [8] and its intertwined relations with the political, legal, economic and cultural dimensions of countries, societies and target populations, calls upon researchers to leverage resources from an extensive array of fields.

Second, as the ultimate outcome of healthcare consultations is often a socially constructed product jointly negotiated among providers, patients and interpreters, researchers often need to take a multi-stakeholder perspective when investigating interpreting activities in healthcare settings, taking into account the opinions of all three parties alike. In this regard, the Model of Bilingual Health Communication established by Hsieh represents a vivid case in point that illustrates the multi-party model of interpreting, since it perceives healthcare interpreting consultations as a ‘goal-oriented communicative activity’ [9] that entails the participation of not only the three parties of service providers, users and interpreters but also other relevant stakeholders at large within the healthcare system as a whole. As a matter of fact, family member of patients and other health professionals, in some cases, are also involved, exerting their impacts, consciously or unconsciously, on interpreter-mediated cross-language medical consultations with important implications [9], and this finding has been corroborated by recent studies [10-13].

Third, different types of healthcare interpreters bring different impacts on provider-patient communications and influence strategies adopted by providers when they cooperate with professional or non-professional interpreters. It has been identified in the literature of healthcare interpreting studies that conflicting findings exist regarding patients’ experience of interpreting services [14–16], and it has been argued that such conflicting findings could be explained by the fact that previous researchers did not pay enough attention to the types of interpreters involved [8]. Since it has been pointed out, types of interpreters have been investigated in healthcare settings, with some studies demonstrating that non-professional interpreters, in certain cases, are as competent as their professional peers, and even outperform professional interpreters at certain tasks [17–19]. These findings suggest that when investigating healthcare interpreting, researchers should avoid assumptions on the superiority of professional interpreters and seek to secure evidence that demonstrates the influences of different types of interpreters on the outcome of medical encounters [9].

An additional challenge confronting healthcare interpreting research is brought by an emerging trend – patient empowerment and participation – in the research and practice in the healthcare field, a trend that is rising swiftly to prominence and shaping healthcare interpreting studies. Hsieh, for example, through the analysis of audio-recorded consultations mediated by interpreters, pointed out that interpreters, by ‘making inexplicit information explicit and providing the patient with the means of self-advocacy’, contributed to patient empowerment and facilitated patient participation [20] in another research conducted by the same author, in-depth analysis of interviews and focus group discussions with medical service providers was able to identify factors influencing doctors’ choices of interpreters, among which the factor of ‘alliances of care’ – a dimension on the ‘management of patient empowerment and patient receptiveness’ – shaped providers’ consideration of interpreter selection. Specifically, patient empowerment, as understood by providers interviewed in the research, was mainly ensured by informational accuracy, which is assumed to be better achieved by professional interpreters, resulting in providers’ preference for professional interpreters when this factor dominated their consideration [21] in contrast, in a more recent research focused on informal interpreting in the context of immigration, interviews with Turkish-Dutch immigrant patients demonstrated that immigrant patients trusted informal interpreters more than professional interpreters because the former would instill into them a sense of empowerment by often assuming the role of ‘advocates and caregivers of the patients’, and acting ‘in their best interests’ [22].

Obviously, the connotations and implications of patient empowerment could be understood differently from the perspectives of different stakeholders, imposing an extra layer of complexity in the investigation of healthcare interpreting. On one hand, as patients are increasingly involved in decision-making, their voices need to be valued in healthcare interpreting research; on the other hand, opinions from diverse parties in a joint medical consultation should be reflected in order to obtain a more nuanced understanding of patient’s needs and expectations.

In the light of the unique features of healthcare interpreting practice and challenges facing researchers, conducting bibliometric research in this important sub-discipline is needed. As illustrated above, the frontline situations of healthcare interpreting practice differ from country to country, so are the practices involved in various specialties and sub-types. Correspondingly, researchers focus on different themes and employ multiple research methods in their investigation. What is lacking in this field is a panoramic view that can put these situations, practices, themes and methods in perspective, identify their similarities and differences, analyze their strengths and weaknesses and describe the evolution and status quo of healthcare interpreting studies in different socio-political contexts in a systematic manner from a macro level. Moreover, bibliometric studies that encompass all types of research, though quintessential as a compass guiding new comers to the field, could not offer a more in-depth view of the intricacies hidden in different sub-fields of interpreting. That entails the necessity to investigate such sub-fields by mapping relevant studies conducted in the corresponding field so far and identifying emerging trends and suggesting future directions.

## Data collection and analysis

The year 2007 marks an important milestone in healthcare interpreting studies, since it has witnessed the release of ground-breaking standards of practice and the publication of trend-setting articles and books on healthcare interpreting. In this year, the Medical Interpreting Standards of Practice developed by the International Medical Interpreters Association was officially released; Angelelli, a frontrunner in healthcare interpreting studies, published her work in medical interpreters assessment [23] *Healthcare Interpreting: Discourse and Interaction*, co-edited by Pöchhacker and Shlesinger, was published, and this volume, as its editors remarked, is ‘the first-ever collection of research on healthcare interpreting’ [24]. Since then, ever-growing research in healthcare interpreting has borne fruits in the last decade.

Therefore, selecting 2007–2017 as the timeframe, the author has conducted research in four major databases, namely, John Benjamins, Taylor & Francis, Web of Science and Ebsco, targeting journal articles with ‘Medical Interpreting’, ‘Healthcare Interpreting’ or ‘Mental Health Interpreting’ in their titles. With the latest Social Science Citation Index and the Arts & Humanities Citation Index as the reference, only articles that have been published in journals belonging to either or both of the two Indexes are selected while other articles, conference proceedings, book chapters, PhD and MA theses, online articles and news pieces are excluded.

As a result, a total of 40 journal articles have been selected from 23 SSCI or A&HCI journals. It is worth noting that not all the 23 journals are directly related to translation or interpretation. As a matter of fact, only 6 of them are translation and interpreting journals, while 9 journals are related to healthcare studies, and another 8 of them are about linguistics and intercultural communication.

A bottom-up analysis has been made to describe and categorize the selected articles, with a particular focus on their year of publication, authors and affiliated institutes, geographic distribution, titles, abstracts, keywords, purposes, objectives, and methods. Then, a top-down approach has been taken to synthesize articles with similar themes and methods so as to offer general trends that can be perceived in the field.

## Findings

### An overview of the journals

As shown in Table 1 below, the fact that contributors to healthcare interpreting studies come from diverse academic backgrounds underscores the interdisciplinarity of this field, which implies an extensive range of both possible research themes and research paradigms, reflecting wide interests from researchers with various expertise and in different healthcare specialties. It also points to a likely future in which the multi-disciplinary trend of healthcare interpreting studies prevails.

**Table 1.** Composition of the journals.

Categorization of Journal	Name of Journal	Indexes	Number of Articles (2007–2017)
Journals on Translation and Interpreting	Interpreter and Translator Trainer	SSCI, A&HCI	6
	Interpreting	SSCI, A&HCI	4
	Babel	SSCI, A&HCI	2
	Translation and Interpreting Studies	SSCI, A&HCI	3
	The Translator	SSCI, A&HCI	2
	Perspectives	A&HCI	1
Total	6		18
Journals on Healthcare Studies	Patient Education and Counseling	SSCI	3
	Journal of Mental Health	SSCI	2
	Medical Education Online	SSCI	2
	BMC Medical Education	SSCI	1
	Health Affairs	SSCI	1
	Advances in Nursing Science	SSCI	1
	Sexual and Relationship Therapy	SSCI	1
	Ethnicity & Health	SSCI	1
	South African Journal of Psychology	SSCI	1
Total	9		13
Journals on Linguistics and Intercultural Communication	Language and Intercultural Communication	SSCI, A&HCI	2
	Journal of Pragmatics	SSCI, A&HCI	1
	Applied Linguistics Review	SSCI	1
	Language Policy	SSCI, A&HCI	1
	Research on Language and Social Interaction	SSCI	1
	Journal of Applied Communication Research	SSCI	1
	Communication Monographs	SSCI	1
	Journal of Language and Politics	SSCI, A&HCI	1
Total	8		9
Aggregate	23		40

### Thematic analysis

**Figure 1.**
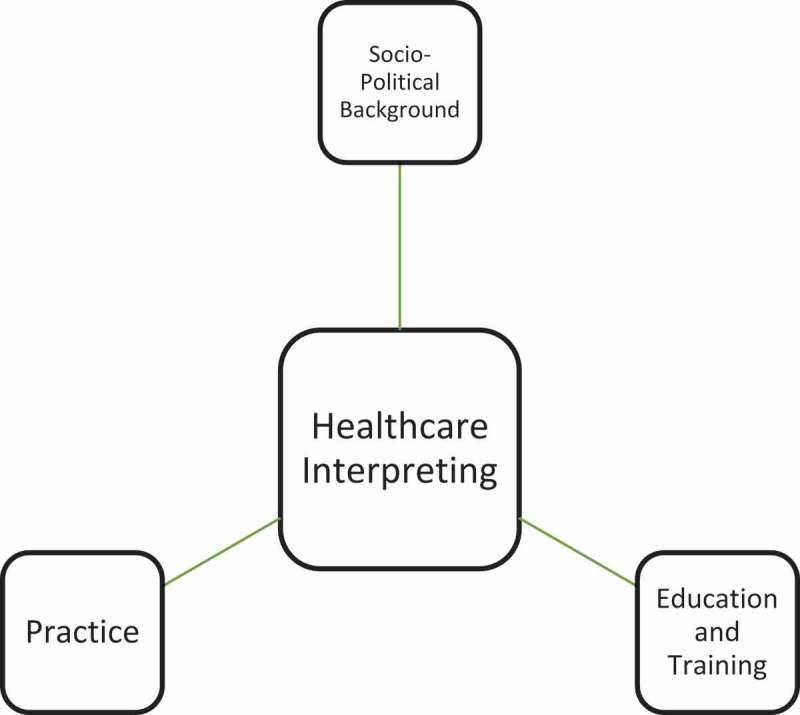
General categories of current research on healthcare interpreting.

**Figure 2.**
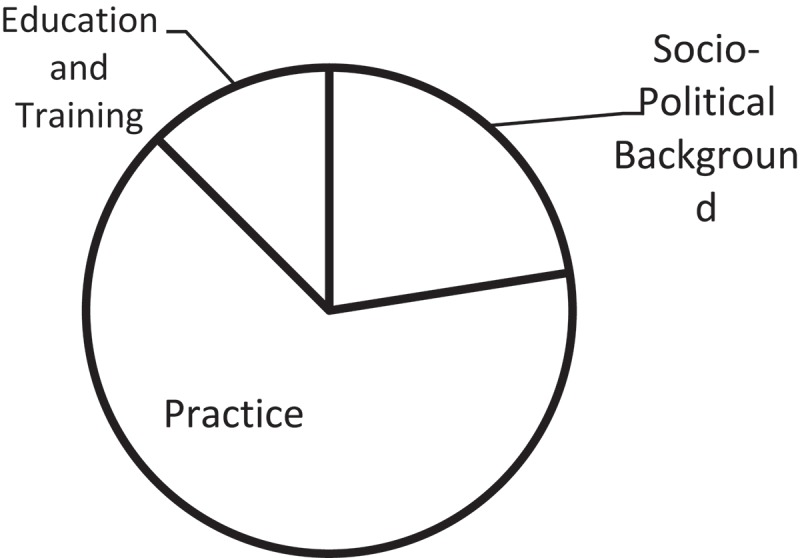
Proportion of articles by theme.

Figures 1 and Figure 2. below have shown the thematic composition and proportion of articles selected in this research. Based on this initial classification and the four-tired categorization adopted by Yan [25], in her attempt at mapping interpreting studies, a three-tiered categorization has been proposed, as illustrated in Table 2 below.

**Table 2.** Detailed breakdown of article categories.

General Themes	No.	Sub Themes	No.	Specific Topics	No.
A. Socio-Political Background	9	a). Language Policy and Access			6
		b). Best Practices and Guidelines			2
		c). History			1
B. Practice	26	a). Product-Oriented	2	1). Genre	1
				2). Source-Target Correspondence	1
		b). Function-Oriented	24	1). Non-professional interpreting	4
				2). Gaze, Positioning and Spatial Orientation	2
				3). Turn-taking and Sequence Organization	2
				4). Emotional Management	3
				5). Role of Interpreter	3
				6). Provider-Interpreter Dynamics	4
				7). Mediation & Brokerage	3
				8). Communication Management and Co-Construction	3
C. Education and Training	5	a). For Interpreting Students			2
		b). For Medical Students			3

It needs to be pointed out that in categorizing the sub-themes of research on healthcare interpreting practice, the concept of ‘product-oriented’ and ‘function-oriented’ research has been borrowed from Holmes’ seminal paper on ‘The Name and Nature of Translation Studies’, in which the product-oriented translation research focused on the description or comparison of translated texts, while the function-oriented translation research was ‘a study of contexts rather than texts’, since it was more interested in the functions of translation in the ‘recipient socio-cultural situation’ [26]. Therefore, the topics of the genre and source-target correspondence have been put under the sub-category of product-oriented research, and other topics taken from a sociological and cultural perspective have been grouped under the sub-category of function-oriented research.

#### Articles on socio-political background

##### Language policy and access

Language policy and access is a frequently researched topic in healthcare interpreting studies. Articles on this topic mainly focus on the access to interpreting services and quality healthcare services in different countries and regions, coupled with the introduction to the evolution of laws and policies promulgated by the government and legislative bodies, which had their own impacts on the industry and practice of language services for the healthcare sector. The guarantee of social justice and human rights for the general public, and for the immigrant population and refugees in particular, has been a major concern and highlight for those papers. A case in point would be Lee’s attempt to map out the landscape in healthcare interpreting in South Korea with a focus on exposing the problems in medical consultation involving patients with limited proficiency in Korean – and thus mediated by interpreters [27]. According to this study, most medical service providers in Korea speak English directly to their patients regardless of the linguistic preferences of the service recipients, leading to low patients’ satisfaction with language access. In a response to the current and potential challenges related to language access facing Korean hospitals, the author has put forward several suggestions, calling for legislative, administrative, educational and financial efforts to be made to ensure the provision of adequate technological and human resources to meet the growing demand of healthcare interpreting services in Korea [27]. On a broader level, these suggestions transcend national borders and could be potentially drawn upon by other countries facing the same set of challenges in their healthcare sectors. From an academic perspective, other scholars could also investigate their own countries or countries they are interested in regarding the status quo and existing problems in the provision of interpreting services and come up with their own insights concerning country-specific solutions to those problems identified.

##### Best practices and guidelines

Best practices and guidelines, another important topic, is more concerned with the interpreting and healthcare industry, providing solutions to the conflicts and tensions existing between patients, providers, and interpreters when there is a lack of coordinated interaction. For example, Tribe and Lane, based on an extensive review of relevant literature have proposed a series of recommendations on how medical service providers should navigate mental health settings in collaboration with interpreters [28]. They have categorized the positive practices into four major categories, including general suggestions applicable to both healthcare professionals and interpreters, as well as practices recommended before, during and after each medical session. On a general level, a medical agency should conduct a needs assessment to ascertain whether a genuine need for interpreting services exists; moreover, special care should be taken to provide support for interpreters who might experience vicarious trauma. In the preparatory stage of a session, efforts should be made to ensure, as much as possible, that the interpreter and the patient share similar linguistic, ethnic and religious backgrounds; it is also recommended that service providers spend some time before each session to brief interpreters on session objectives, contextual and cultural information, as well as terminologies; room organization and chairs arrangement merit thoughtful consideration, where a triangular positioning among the three parties in an interpreter-mediated consultation seems to be ideal. Within a clinical consultation, service providers are recommended to refrain from using ‘proverbs, sayings and colloquial language’ or ‘complicated technical language’ [28]. In addition, speakers ought to control their speech rates in an appropriate range and divide their speech into segments that are easy to follow, memorize and interpret. After the session, healthcare service providers are assumed to have an obligation to provide continued care for interpreters by a short debriefing session focusing on a review and evaluation of their collaboration. Such recommendations on best practices in healthcare interpreting would serve as a viable foundation for more systematic codes of conduct or ethics to be formulated accordingly, and a possible direction in future would be to extend such recommendations from a certain specialty to other clinical contexts and strive towards the full coverage of a comprehensive spectrum of healthcare specialties in which different specialties are underpinned by their corresponding recommendations on best practices, or better even, codes of conduct.

##### History

The historical dimension of healthcare interpreting has been rarely researched, with only one article selected in this study, delving into a distinctive combination of healthcare interpreting and asylum interpreting in Germany in the early 20^th^ century [29]. By examining the unique situation in which multilingual patients with mental health problems served as interpreters for their fellow patients in an asylum for ‘insane re-migrants’, the research demonstrated that the patients-turned interpreters took an active part in shaping the psychiatric therapies and building the medical archives for their fellow patients. Despite their own mental health conditions, the interpreters assumed the role of opinion provider and diagnostic consultant, exercising considerable freedom in the asylum, contributing to its functional operation. The findings of this research, though based on historical archives distant from the contemporary era, are highly relevant to today’s interpreting practices in healthcare settings and offers much food for thought. When fellow patients come to serve as interpreters for their peers, the previous discussion about different types of interpreters, along with their impacts on provider-patient communication and providers’ strategies in collaborating with them, is enhanced with historical evidence, illustrating possible new areas in which the role boundaries between patients and interpreters could be further investigated.

#### Articles on practice

**Figure 3.**
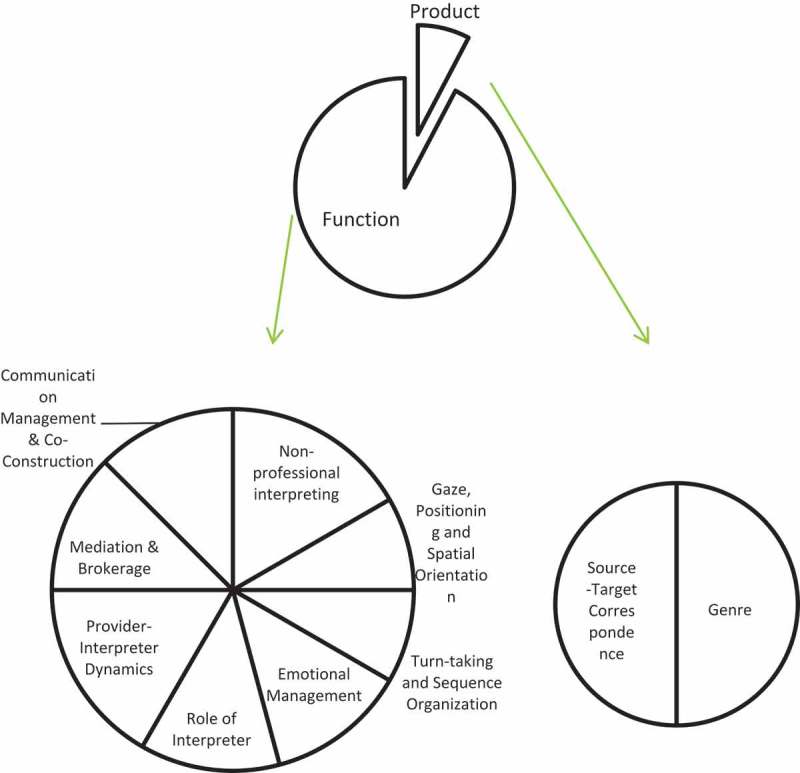
Proportion of articles on healthcare interpreting practice by sub-categories.

Figure 3 below has demonstrated the sub-categories and their proportion in the general category of practice.

##### Non-professional interpreting

Specifically, non-professional interpreting, or ad-hoc interpreting, has been a highly frequent theme in the research on healthcare interpreting practice. Articles in this sub-field have pervasively pointed out the status quo where there exists a lack of professionally trained interpreters in healthcare settings, which, as a result, has led to the situation in which non-professional interpreters, such as nurses, patients’ relatives, healthcare workers, come to serve as interpreters in consultations on an ad-hoc basis. Schouten and his colleagues, for instance, have set out to compare the practice of informal interpreting in two European countries from a socio-cultural perspective by conducting in-depth interviews [30]. Their findings suggest that informal interpreters tend to deviate from the norms upheld by professional interpreters who are trained to serve as a mere conduit and thus become invisible in a communicative event. What they exhibit proves to be quite the opposite: they would commonly advocate for the rights and interests of the patients by message manipulation. Another set of significant findings from the research is derived from a comparison and contrast between the two countries focused upon – the Netherlands and Turkey, and mainly indicate the differences in the attitudes and values involved regarding informal interpreting between interpreters from the two countries. As pointed out by the authors, both the inter-lingual encounters and the participating stakeholders are conditioned and shaped by ‘socio-political and cultural contexts’ [30], Turkish interpreters have demonstrated a more active attitude in providing interpreting services for their elders compared with their Netherlands counterparts, which, according to the authors, could be associated with the individualistic vis-à-vis collectivistic dichotomy perceived as prevalent in the Netherlands and Turkey, respectively. The association of interpreters’ attitudes with the socio-political contexts of countries and the comparison between interpreters from different countries offers fresh insights into the norms, values, and beliefs held by non-professional interpreters, which, to a certain extent, determine the working practices of interpreters. While socio-political contexts serve as fertile soil in which interpreter behaviors are rooted, a comparative perspective targeting two or more groups of interpreters of distinctive cultural imprints and traditional legacies reinforces the close relationship between interpreters’ practices and their cultural identity.

##### Emotional management

Emotional management, a topic of special significance to the health of healthcare interpreters and to the maintenance and sustainability of a healthcare consultation, is another sub-theme in the third category. It is generally acknowledged by interpreting researchers that interpreters working in refugee or healthcare settings, in which human suffering is constantly exposed to interpreters, can have potential psychological and physical impacts on interpreters, and in some cases, even leave interpreters with precarious trauma. A representative research in this regard would be Green’s exploration of how Kurdish interpreters worked in mental health settings in the UK. Based on the findings, the author recommended that ‘training on mental health issues and self-care’ be provided to interpreters working in such conditions [31]. Increasing awareness on the often-precarious working conditions facing healthcare interpreters makes it imperative for researchers to explore the many possible hazards threatening the physical and mental wellbeing of interpreters, describe them in fuller details and investigate how these sources of traumatic experiences impact interpreter performance and how measures could be taken to minimize interpreters’ exposure to such risks.

##### Provider and interpreter dynamics

Provider and interpreter dynamics is a frequently discussed sub-theme in healthcare interpreting studies. Researchers interested in this regard have explored the ways in which interpreters could work better with healthcare providers, and suggest that both have their pre-existing assumptions and positions on their respective roles in an actual communicative event, and move along a spectrum of different roles. It is believed that both parties should, through negotiation and collaboration, identify an ideal balance and alignment of their expectation on each other’s role, which could improve the outcome of healthcare services. In this aspect, Galvan’s work represents a recent effort towards building an increasingly constructive environment that facilitates interpreter-mediated medical consultations [32]. Among the guidelines and solutions the author proposed, building trustworthy relations, communicating information adequately and reaching consensus among all stakeholders involved in a medical consultation have been highlighted as particularly relevant [32]. Studies in this sub-theme are conducive in establishing dynamic benchmarks against which both interpreters and providers position themselves according to a set of expectations and norms, and facilitate the concerted efforts by both parties towards the attainment of improved and satisfactory healthcare outcomes.

Importantly, the field of healthcare interpreting research is highly contextualized, which is reflected by the fact that some articles selected in this research have explicitly indicated the specific healthcare specialties where the object of the study – often interpreter-mediated communicative events, took place. Therefore, this explicitation of healthcare specialties should be regarded as a significant and relatively unique feature for studies on healthcare interpreting, regardless of whether they belong to the category of the socio-political background, practice, and education and training. Table 3 below has listed some of the most frequently researched specialties, albeit half of the selected research has not specified the specialties.

**Table 3.** Frequency of sub-specialties.

N	Sub-Specialties	Freq	%
1	Unspecified	20	50
2	Psychiatry (Mental Health)	10	25
3	Gynecology	3	7.5
4	Pediatrics	3	7.5
5	Oncology	2	5.0
6	Nursing	1	2.5
7	Chronic Diseases	1	2.5

#### Articles on education and training

In this category, McEvoy aimed at instructing medical students about how to cooperate with interpreters by involving interpreters in an educational workshop designed to raise the cultural awareness of medical students, and arrived at the conclusion that interpreter positioning and assuring confidentiality were problematic, both of which should be enhanced in training to improve inter-lingual healthcare consultations [33]. In contrast, Ono adopted the method of systematic literature review and aimed at identifying the ‘core competencies’ of medical interpreters, including ‘(1) maintaining accuracy and completeness; (2) medical terminology and the human body; (3) behaving ethically and making ethical decisions; (4) nonverbal communication skills; and (5) cross-cultural communication skills’ [34].

The different perspectives focusing on medical students and interpreting students, respectively, showcases the mutual interest in the fields of medical studies and interpreting studies, as well as the inter-disciplinary nature of healthcare interpreting studies. In future, more integrated research design, drawing upon the expertise and experience of both fields, is expected to generate more comprehensive insights into a range of possibilities in the collaboration between providers and interpreters.

**Figure 4.**
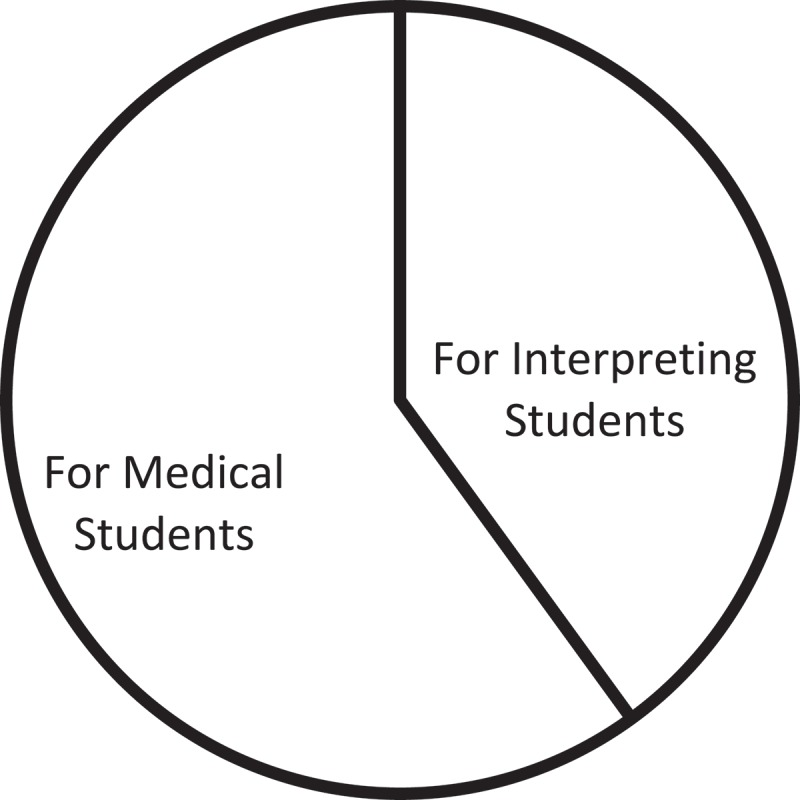
Proportion of articles on education and training by sub-categories.

It is worth noting that articles on education and training for medical students outnumber those for interpreting students, as illustrated in Figure 4 below.

### Methodological analysis

**Figure 5.**
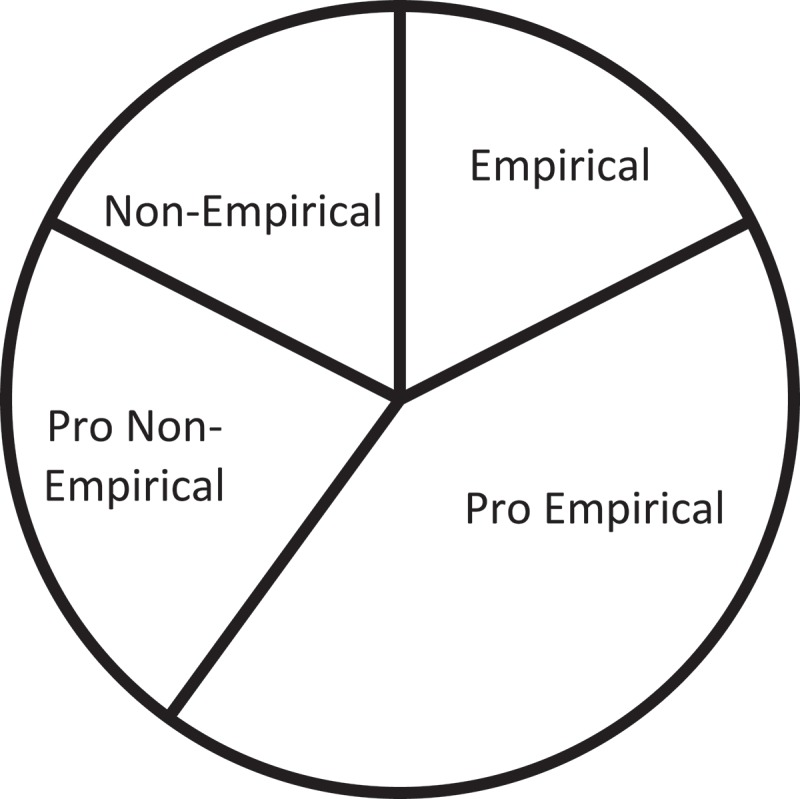
Proportion of articles categorized in different research methods.

A three-tiered system of categorization, covering a spectrum from empirical to non-empirical approaches, is established to categorize the research methods employed, as shown in Table 4 below, while Figure 5 demonstrates the proportion of those approaches.

**Table 4.** A detailed breakdown of research methods.

Research Method	No.	Method Orientation	No.	Method of Data Collection	No.
A. Empirical	24	a). Pure Empirical	7	1). Observation	3
				2). Experimentation	4
		b). Pro Empirical	17	1). Questionnaire Survey	7
				2). Interview	7
				3). Focus Group	3
B. Non-Empirical	16	a). Pro Non-Empirical	9	1). Case Study	3
				2). Corpus-Based	6
		b). Pure Non-Empirical	7	1). Systematic Literature Review	7

#### Pure empirical

Purely empirical studies are defined as those that adopt the methods of observation or experimentation. For example, Zhan and Zeng have resorted to observation to examine the degree of visibility of interpreters working in a hospital based in southern China [35]. In the same vein, Krystallidou has conducted a training experiment to examine the ‘direction of gaze and body orientation’ of interpreters and other participants in medical consultations so as to demonstrate the effects of non-verbal communication in healthcare interpreting. This experiment produced sound evidence supporting the significance of non-verbal communication, which may contribute to shaping the future model of healthcare interpreter training [36].

#### Pro-empirical

For pro-empirical studies, commonly selected research methods include questionnaire survey, interview, and focus group. Among them, questionnaire survey and interview are most frequently adopted as the research methods for articles on healthcare interpreting practice. For instance, Bischoff has resorted to questionnaire survey to investigate interpreters’ self-perception regarding their services in the macro-environment of immigration and the microenvironment of the healthcare industry [37]. Likewise, Zimanyi interviewed mental healthcare service providers and interpreters and delved into the issue of communication control in interpreter-mediated encounters between patients and doctors [13]. Another important approach in the pro-empirical category is the method of conducting a focus group, which essentially involves the organization of participants in a joint and often in-depth discussion of a pre-designed topic, through which patterns of communication could be identified. In this connection, Angelelli, relying on the method of the focus group, has designed a battery of tests which were believed to gauge the competency of medical interpreters [23]. Focusing on language proficiency and interpreter readiness, the set of tests comprise a language proficiency test and an interpreter readiness test, the testing materials of which have been based upon authentic events in the realm of public services involving the mediation of interpreters and thus reflect the reality of interpreting in healthcare settings. The set of tests are useful to interpreting instructors inasmuch as the interpreter readiness test can help diagnose the current level of interpreting capability of students and place them in appropriate programs accordingly, and the language proficiency test can identify whether prospective students need to attend language enhancement classes and how they are performing in such enhancement programs. Ultimately, both tests, as argued by the author, could help filter and select potential candidates applying for healthcare interpreting training programs.

#### Pro non-empirical

For pro-non-empirical studies, case studies and corpus-based studies have become the mainstream in the last decade. A prominent feature of such studies is that the data in the research is not generated on the ground, as is the case for most empirical and pro-empirical studies, but originated from pre-existing databases or research programs, which serve as a natural reservoir of rich resources at the disposal of researchers. Baraldi and Gavioli, for instance, have built a corpus based on literature accumulated over a long period of time to shed light upon the work of ‘intercultural mediators’ who serve as healthcare interpreters [38].

#### Pure non-empirical

Contrary to the intuition of some, quite a few articles on the practice of healthcare interpreting choose to conduct the study in a purely non-empirical fashion, that is, adopting the method of systematic literature review. Such an approach has been preferred in studies of the socio-political background of healthcare interpreting, since relevant laws, policies, regulations, and guidelines are mainly explored from existing literature. Hsieh and Nicodemus, for instance, leveraged database resources and online academic search engines in an attempt to review relevant research focusing on how emotions of participants shaped the outcome of interpreter-mediated medical consultations and how such emotions ought to be managed [39].

#### Integrating themes with methods

**Figure 6.**
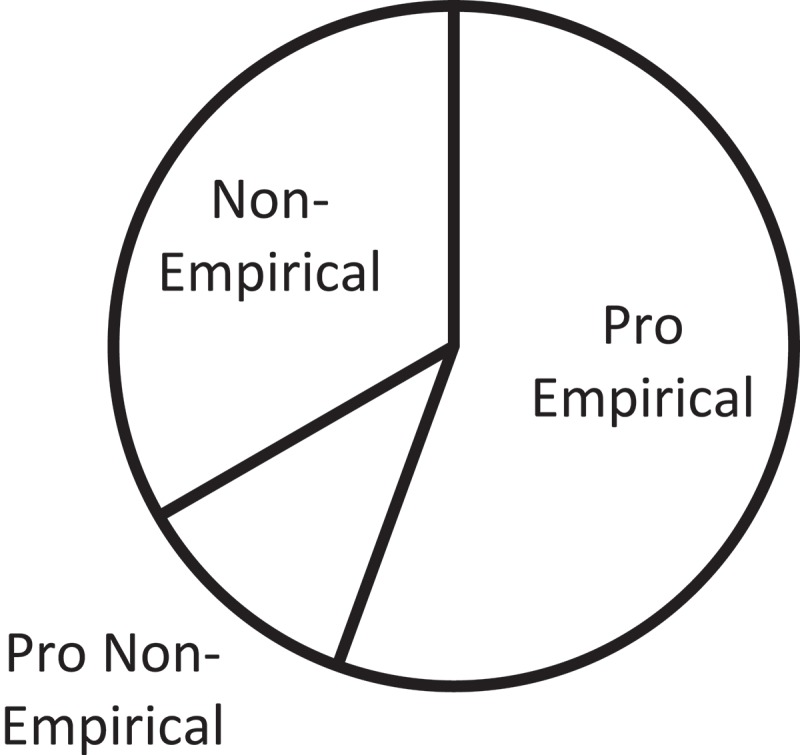
Research methods in articles on language policy and access.

If the thematic perspective is integrated with the methodological analysis, then the methodological composition of articles on language policy and access can be identified, as shown in Figure 6, which indicates that pro-empirical methods are the mainstream for this category of the theme. This means that the methods of questionnaire survey, interview, and focus group are most frequently employed in the study of language policy and access in healthcare settings. A possible explanation for this particular tendency is that there exists a quasi-vacuum in many countries regarding the provision of language access – and interpreting services in particular – in healthcare settings, making it necessary to enlarge the scope of research in an initial stage to a region-wide or even nation-wide level for a sufficient exploration and collection of on-the-ground data regarding the political and legal context of language policies and access. Obviously, among the commonly used research methods in humanities and social sciences, questionnaire survey, categorized as belonging to the pro-empirical group in this paper, stands out as being able to amass a large quantity of data efficiently, thus an ideal choice of method for research on language policy and access.

Nevertheless, the concentration of pro-empirical methods employed in this sub-theme of research also points to the lack of other alternatives, namely, case studies, corpus-based studies, and systemic literature review. It is believed that wide-reaching research scope would better serve its purposes when combined with focused research design, and case studies would be constructive in helping researchers to zoom in on specific institutions, locations, and target populations intimately involved in healthcare settings. Moreover, while questionnaire surveys provide important first-hand data, a thorough review and analysis of existing databases, corpora and relevant literature would also offer comprehensive and invaluable information concerning the history and status quo of language policies and access in healthcare settings. Therefore, it is suggested that future research in this sub-theme diversify their research methods so as to ensure methodological mutual reinforcement and complementarity. In this connection, the Model of Bilingual Health Communication stands out as it has been proposed by adopting a mixed-method approach and based on data collected from a wide array of channels such as ‘ethnographic data, participant observation, in-depth interviews, focus groups, and surveys’ [9].

**Figure 7.**
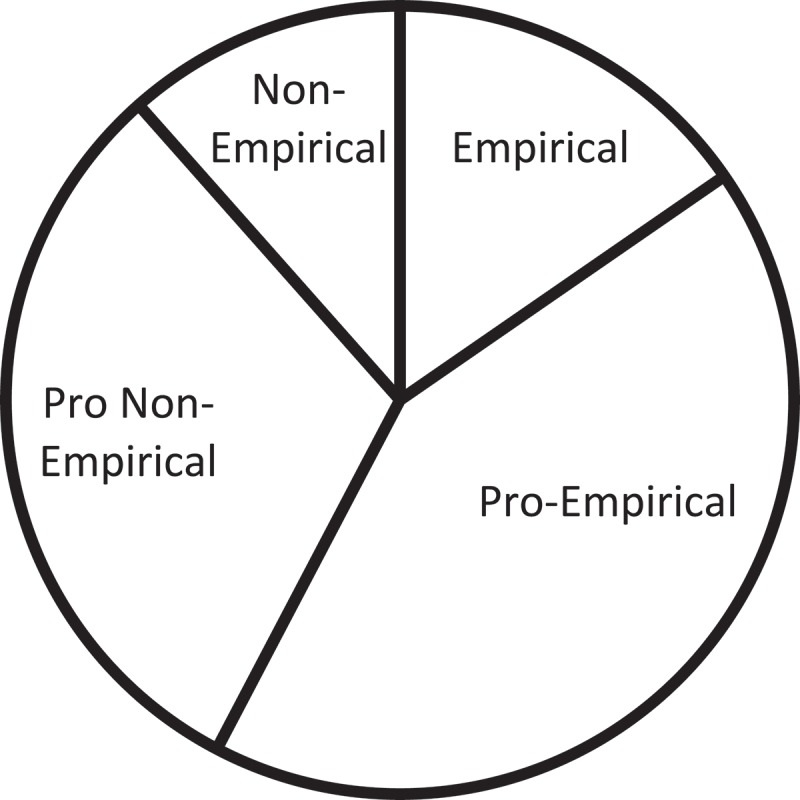
Research methods in articles on practice.

**Figure 8.**
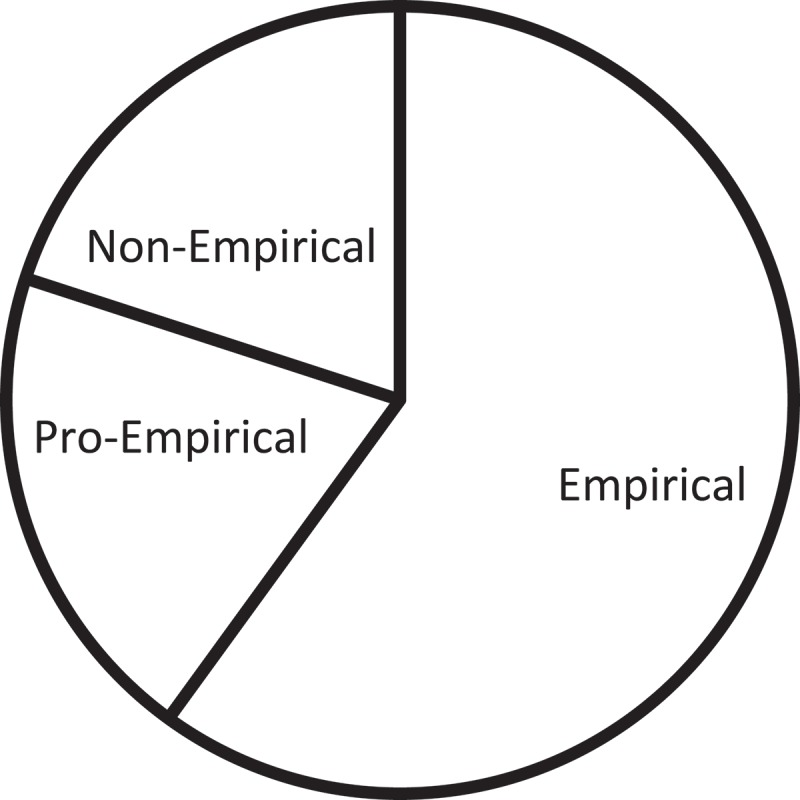
Research methods in articles on education and training.

Likewise, the methodological composition for articles on practice and education and training could also be presented, as demonstrated in the same manner in Figures 7 and Figure 8. Obviously, for articles on the practice of healthcare interpreting, pro-empirical methods take the lead, while pro-non-empirical methods follow closely. For articles focusing on education and training, empirical methods stand out as the dominant approach, and pro-empirical and non-empirical methods share the distribution of the rest of the articles. While questionnaire survey, interview, and focus group methods would indeed prove to be instrumental in serving the purposes of studies on interpreting practice when the research objective is mainly descriptive, the lack of observation and experimentation points to insufficient explanatory and predictive power by such research, indicating the fact that practice-oriented research is still lingering at a relatively insufficient level of academic insights. Likewise, although research in education would benefit from observation and experimentation – two approaches frequently adopted in pedagogical research, attempts at other methods would encourage the development of the mixed-method design which, as an emerging hallmark of healthcare interpreting studies, could enhance the validity and reliability of research results.

## Discussion

First of all, research on healthcare interpreting features a clear combination, but not necessarily integration, of different disciplines including translation and interpreting, healthcare studies and linguistic and intercultural investigations. Thus, both ontological and inter-disciplinary perspectives have been incorporated into this specific field. The shared interest among practitioners, researchers, and educators from the three above-mentioned sectors is evident through the mapping of studies over the past decade.

However, it can be argued that the field of translation and interpreting studies has not realized an organic integration and harmony with the field of healthcare studies. Currently, there seems to be little mutual dialogue between the two. This observation is corroborated by Hsieh, who rightly points out that healthcare interpreting studies, which deserve to be investigated through cross-disciplinary perspectives, are still largely confined to the field of interpreting studies. Related disciplines, especially those closer to medical studies, are left out of the picture, which comprises the depth and comprehensiveness of existing research [9]. As a result, some studies on healthcare interpreting mainly borrowed or introduced the concepts, theories, frameworks, models or paradigms that have been proved to be more mature from the more established field of healthcare studies. Furthermore, some research on healthcare interpreting has been somewhat reduced to a mere target or object of research, while the theoretical framework or the pre-existing perspectives or methodologies still come from either the healthcare studies or community interpreting, which encompasses healthcare settings. This has led to a situation in which the healthcare setting risks being regarded as a mere testing ground for relevant hypotheses or theories generated and proposed from other disciplines.

In this regard, two streams of research borrowing external constructs could be identified. The first stream of research mainly utilizes existing theoretical and analytical frameworks to reach their objective. For example, the theory of Pragmalinguistics and the Cooperative Principle are leveraged in an attempt to offer guidelines for a more positive environment for mental health interpreting [32]; the theory of communication and cross-cultural adaptation have been used to address the issues of conflict in interpreter-mediated mental health settings [40]; the social cognitive theory and role-reversal theory have been adopted to investigate the widely known phenomenon that children sometimes come to play the role of healthcare interpreters with their interpreting competency examined [41]; the theory of vicarious traumatization (VI) has been taken as a perspective to account for the discursive practices of Kurdish refugees serving as mental health interpreters in UK institutional settings [31]. The other stream of research commonly draws upon important individual concepts to inform their research design. For instance, several linguistic concepts, such as discourse structure, contextual variables of field, tenor and mode, the concept of genre and sub-genre have been entertained in the teaching of dialogue interpreting in healthcare settings [42]; the definition and types of explicitation have been foregrounded and highlighted in a novice-expert paradigm focusing on regularities identified in the explicitation strategies adopted by professional interpreters and interpreting students, respectively, [43]; and the concept of visibility and text-ownership have been singled out as important elements in the research object and methodology, respectively, for a longitudinal study targeting a hospital in Southern China, aimed at exploring reasons for interpreters working at the hospital adopting a more-than-mere-conduit position in medical consultations involving non-native patients [35].

This one-directional status quo of communication between T & I and healthcare studies could be further evidenced by the fact that not only the number of journals on healthcare studies selected in this paper is larger than that of journals directly related to translation and interpreting, but also by the phenomenon that more research on the sub-theme of education and training centered around medical students than interpreting students. As a matter of fact, the healthcare field seems to be more concerned with teaching medical students how to work with interpreters than their counterparts are with teaching interpreting students to cooperate with medical service providers. This is further evidenced by the fact that more and more healthcare researchers are starting to conduct research on healthcare interpreting [44-49]. On one hand, such a trend indicates that scholars from the field of healthcare studies have expanded and enriched the field by transcending the scope of interpreters’ ‘discursive practices’ [9] and that the integration between interpreting studies and healthcare studies is being accelerated; on the other hand, it exposes the lack of initiatives on the part of the T & I community regarding the research of healthcare interpreting due to, possibly, the insufficient mastery of healthcare expertise among other factors. Thus, it can be said that interpreting scholars face a higher threshold in their journey towards the healthcare field than the one faced by healthcare scholars when they migrate to the interpreting sector. Therefore, it is hoped and suggested that greater strides forward are taken by interpreting scholars towards the healthcare field by receiving more training in the target field through cross-interdisciplinary endeavors in the form of dedicated workshops and short-term programs aimed at making language professionals and researchers better acquainted with the healthcare landscape.

Secondly, function-oriented research seems to attract much more attention from researchers than product-oriented research, which denotes rising interests in adopting a cross-disciplinary approach in the investigation of healthcare interpreting practice, with a particular focus on socio-cultural perspectives. This is also evidenced by the fact that research on language policies and access constitutes the bulk of the research on socio-political background. In order to fully capture the multi-disciplinary nature of healthcare interpreting studies, it is suggested that future studies on healthcare interpreting incorporate more inter-disciplinary perspectives to shed more light on the socio-political background, filling in the vacuum in countries concerned. A viable approach in future studies would be to leverage the insights from sociologists, legal experts, political scientists, economists and cultural scholars in a joint attempt to pinpoint the macro-level environment in specific countries regarding the provision of interpreting services in healthcare settings. Such multi-disciplinary collaboration could be substantiated through legislation, policy-making and consultation-based agenda-setting, and an integration between the bottom-up and the top-down approach is applicable to all three levels of collaboration. On the legislative level, results from the joint research by interpreting scholars and researchers from related disciplines serve as the evidence driving legislative efforts aimed at improving language access to patients with limited linguistic proficiency, and such improved legal environment, in turn, stimulates further research into healthcare interpreting. On the policy-making level, healthcare interpreting scholars work with language policy researchers in an attempt to shape policy-making in specific countries, and after the research results are utilized to revise and improve relevant policies and regulations, healthcare interpreting scholars and their collaborators will identify new frontier of research. On the agenda-setting level, healthcare interpreting scholars proactively participate in publicity campaigns with the help of medical professionals who have the expertise and knowledge to promote medical science popularization and education, mobilizing more patients to air their concerns and put forward their suggestions to influence public opinions and set the agenda of social debate.

Thirdly, some research on healthcare interpreting has specified the specialties while others have not. As far as the concept of ‘specialty’ is concerned, the unique feature of such a field lies in its diverse healthcare specialties, distinguishing it apart from other settings – legal, educational, religious, etc. – under the general concept of community interpreting or public service interpreting. Thus, it is recommended that future research on healthcare interpreting highlight the specific specialties and fully utilize such concrete specialties as sources for guidelines informing the practice or for possible theories and models most suited to the specialties concerned. Apparently, this calls for a sufficient and detailed description of the specific specialties involved in each case, based on which relevant characteristics of the specialties should be duly reviewed and summarized, laying a solid groundwork for the investigation into interpreting activities in such specialties. Moreover, as one would naturally argue that mental health interpreting involves a rather different scenario from physical health interpreting, researchers are advised to be more familiar with various healthcare specialties and what they involve. The specificity of each specialty would entail vastly different norms and standards related to provider-patient relations, patient needs, pathological idiosyncrasies, and the patterns of collaboration among providers, patients, and interpreters, giving rise to contrasting sets of challenges for all stakeholders. Such challenges, when properly registered, examined and analyzed by researchers, may prove to be promising areas of innovation. For instance, Hsieh [9], identifies differences among various specialties in healthcare settings by highlighting the different attitudes and expectations of stakeholders regarding whether interpreters are patient allies. The findings suggest that service providers from various medical specialties have varying requirements on interpreters in terms of the extent to which they are expected to help service users and uphold their rights and interests [9]. Specifically, medical professionals from the mental health and oncological specialty generally disapprove of interpreters extending their non-linguistic services for patients when an interpreter-mediated encounter is over, while providers from emergency medicine and nursing adopt a more inclusive and supportive attitude toward such practices [9].

When the scope of the healthcare specialties go beyond the collective expertise of the interpreting research community, insightful opinions and suggestions should be solicited from healthcare researchers, healthcare professionals, healthcare interpreters, and even patients. In future, frequent and constructive cross-fertilization and cross-referencing among these groups of stakeholders should be encouraged, in which thorough descriptions of the specialties could be offered by healthcare-related parties, and how these specialties inform and even drive interpreting studies could be established by interpreting researchers. When this bottom-up perspective is taken, endogenous insights from the interpreting practice itself could be generated, thus avoiding the phenomenon where the setting is reduced to a secondary position, only to be used as a background to set off other disciplines.

In addition, the results of this paper show that those studies that specified the specialties mainly concentrate on mental health or psychiatry, which is understandable since the practice of such a specialty mainly relies on the verbal communication between patients and doctors. Yet, it is quite apparent that other sub-specialties have not received sufficient attention from researchers. Therefore, it is recommended that future studies on healthcare interpreting expand more into less explored specialties so that a more comprehensive picture of the field could be rendered.

## Conclusions

Healthcare interpreting has enjoyed robust development in the last decade with an increasing number of publications from scholars in various disciplines, demonstrating the interdisciplinary nature of this field. At the same time, healthcare interpreting studies have branched out into a wide range of themes and methodologies, and delivered fruitful results. This research has mapped healthcare interpreting research over the past decade based on an analysis of articles published on SSCI or A&HCI journals. On top of the thematic, methodological, demographic and geographic analysis of the articles selected in the paper, trends of the research could be perceived and future directions are pointed out.

Upon reviewing the articles selected, several findings have been generated. First, it has been found that journals publishing healthcare interpreting studies covered in this research belong to multiple disciplines, spanning interpreting studies, linguistic and cultural studies, and healthcare studies, reflecting the interdisciplinary development of this field. Second, four major themes have been identified, i.e. the socio-political background, interpreting practice, education and training, and book review, under which diverse sub-themes have been pointed out. Third, a full spectrum of methodologies has been adopted in healthcare interpreting research, ranging from purely empirical designs to purely non-empirical ones, with different layers in between. Fourth, the demographic picture of the studies presents a clear trend in which the USA, followed by other countries mostly in Western Europe, take the lead in healthcare interpreting studies, while there has been a growing concern in this regard in emerging regions as well.

Based on the findings, several observations and recommendations have been made, regarding the status quo and future directions. First, the field of healthcare interpreting studies has been informed by both the T&I studies and healthcare studies, yet the two have not fully integrated with each other, making the more harmonized interaction between the two fields a possible direction in the future. Second, research on the practice of healthcare interpreting remains the dominant theme in the field, suggesting that there remains a vacuum of macro-level socio-political support in the form of laws, policies, and regulation in some countries. This leads to another possible direction which points to increasing interest in combining sociological, political, economic and historical perspectives in this field. Third, while currently many studies have not specified the specific sub-specialties involved, it is suggested that future research would highlight the sub-specialties so as to reflect the unique features of healthcare interpreting and that more research would explore sub-specialties that are less touched upon. In this regard, multi-stakeholder collaboration has been encouraged as a viable framework to ensure that future research could be specialty-based, informed and driven, and organic theoretical and analytical framework could be fostered from the ground up.
